# Effect of deep learning-based assistive technology use on chest radiograph interpretation by emergency department physicians: a prospective interventional simulation-based study

**DOI:** 10.1186/s12911-021-01679-4

**Published:** 2021-11-08

**Authors:** Ji Hoon Kim, Sang Gil Han, Ara Cho, Hye Jung Shin, Song-Ee Baek

**Affiliations:** 1 Department of Emergency Medicine, Yonsei University College of Medicine, 50 Yonsei-ro, Seodaemun-gu, Seoul, 03722 South Korea; 2 Department of Preventive Medicine , Yonsei University College of Medicine, 50 Yonsei-ro, Seodaemun-gu, Seoul, 03722 South Korea; 3 Department of Research Affairs, Biostatistics Collaboration Unit, Yonsei University College of Medicine, 50 Yonsei-ro, Seodaemun-gu, Seoul, 03722 South Korea; 4 Department of Radiology, Division of Emergency Radiology, Yonsei University College of Medicine, 50 Yonsei-ro, Seodaemun-gu, Seoul, 03722 South Korea

**Keywords:** Chest radiograph, Emergency department, Deep learning-based assistive technology, Decision-making

## Abstract

**Background:**

Interpretation of chest radiographs (CRs) by emergency department (ED) physicians is inferior to that by radiologists. Recent studies have investigated the effect of deep learning-based assistive technology on CR interpretation (DLCR), although its relevance to ED physicians remains unclear. This study aimed to investigate whether DLCR supports CR interpretation and the clinical decision-making of ED physicians.

**Methods:**

We conducted a prospective interventional study using a web-based performance assessment system. Study participants were recruited through the official notice targeting board for certified emergency physicians and residents working at the present ED. Of the eight ED physicians who volunteered to participate in the study, seven ED physicians were included, while one participant declared withdrawal during performance assessment. Seven physicians’ CR interpretations and clinical decision-making were assessed based on the clinical data from 388 patients, including detecting the target lesion with DLCR. Participant performance was evaluated by area under the receiver operating characteristic curve (AUROC), sensitivity, specificity, and accuracy analyses; decision-making consistency was measured by kappa statistics. ED physicians with < 24 months of experience were defined as ‘inexperienced’.

**Results:**

Among the 388 simulated cases, 259 (66.8%) had CR abnormality. Their median value of abnormality score measured by DLCR was 59.3 (31.77, 76.25) compared to a score of 3.35 (1.57, 8.89) for cases of normal CR. There was a difference in performance between ED physicians working with and without DLCR (AUROC: 0.801, *P* < 0.001). The diagnostic sensitivity and accuracy of CR were higher for all ED physicians working with DLCR than for those working without it. The overall kappa value for decision-making consistency was 0.902 (95% confidence interval [CI] 0.884–0.920); concurrently, the kappa value for the experienced group was 0.956 (95% CI 0.934–0.979), and that for the inexperienced group was 0.862 (95% CI 0.835–0.889).

**Conclusions:**

This study presents preliminary evidence that ED physicians using DLCR in a clinical setting perform better at CR interpretation than their counterparts who do not use this technology. DLCR use influenced the clinical decision-making of inexperienced physicians more strongly than that of experienced physicians. These findings require prospective validation before DLCR can be recommended for use in routine clinical practice.

## Background

Chest radiography is a basic imaging test for thoracic disease, accounting for 26% of all diagnostic radiology tests performed in this field [1–8]. It is estimated that 9–10% of patients present at the ED with respiratory complaints, suggesting that the demand for chest radiography in this context is particularly high [9]. However, CR interpretation is a difficult task that requires both experience and expertise because various anatomical structures tend to overlap when captured on a single two-dimensional image, different diseases may have a similar presentation, and specific diseases may present with different characteristics [10]. Therefore, CR interpretation is associated with a high error rate, previously estimated at 22% [11]. Moreover, prior studies have reported that CR interpretation by ED physicians is inferior to that by expert radiologists [12–15]. Particularly, in cases of critically ill patients requiring rapid CR interpretation, ambiguous findings may be overlooked, which negatively affects patient safety [16]. The American College of Radiology recommends that an experienced radiologist should interpret the results of all diagnostic radiology tests performed within the ED [17]. However, this recommendation is associated with practical limitations, as coverage by radiologists tends to be restricted during nights and weekends. In fact, a 2014 survey revealed that 73% of radiology departments in the United States did not provide a night-time service [18]; therefore, CR interpretation in the ED setting becomes the responsibility of ED physicians.

Recent studies have reported that CR interpretation using a deep learning-based assistive technology (DLCR) is more accurate than that performed by a reader (or radiologist) alone [19, 20]. However, only a few previous studies have examined the effectiveness of DLCR in clinical practice. Moreover, to the best of our knowledge, no prior study has reported the influence of DLCR use on clinical decision-making. This study investigated whether DLCR aids physicians in performing CR interpretation in clinical practice and whether it affects their clinical decisions.

## Methods

### Study design and participants

We conducted a prospective interventional study using a web-based performance assessment system. The study protocol was reviewed and approved by the Institutional Review Board of Severance Hospital, South Korea (approval number 2019-3134-001) and adhered to the ethical standards of the Declaration of Helsinki. Study participants were recruited through the official notice throughout January 2020. The inclusion criteria were as follows: (1) age > 18 years and (2) board-certified emergency physicians or residents receiving emergency medicine training working at the ED of study site. The exclusion criteria were as follows: (1) those who cannot read the research consent form or do not understand the contents and (2) those who agreed to participate in the study but later withdrew. At that time, there were 10 board-certified emergency physicians and 29 residents receiving emergency medicine training working in this ED. Among them, eight ED physicians volunteered to participate in this study, while one participant was excluded from the study. This participant was a board-certified ED physician with 59 months of experience who withdrew the participation during the performance assessment. Finally, a total of seven participants were included in the study. The participants consisted of two ED physicians with 11 months of experience, two ED physicians with 23 months of experience, one ED physician with 35 months, and one ED physician with 47 months of experience, and two board-certified (emergency medicine) ED physicians with 59 months of experience. The mean age of the participants was 29.6 years, and three ED physicians were female. ED physicians with < 24 months of experience were defined as ‘inexperienced’. We provided all participants with information on the study purpose and simulation system mechanics. Informed consent was obtained from all participants before study enrolment.

### Collection of clinical data used for performance assessment

A total of 411 consecutive patients underwent both chest radiography and chest computed tomography (CT) in September 2019 at a tertiary ED in South Korea, which had more than 100,000 annual visits. For simulation, patient data were extracted from electronic medical records. Images from 23 patients that did not involve any of the three targets (lung nodule, consolidation, and pneumothorax) of DLCR used in this study were excluded. Finally, the clinical data from a total of 388 patients were used for performance assessment. These data were automatically collected through the clinical research analysis portal developed by our medical information department.

### The protocol of simulation sessions for performance assessment

**Fig. 1:**
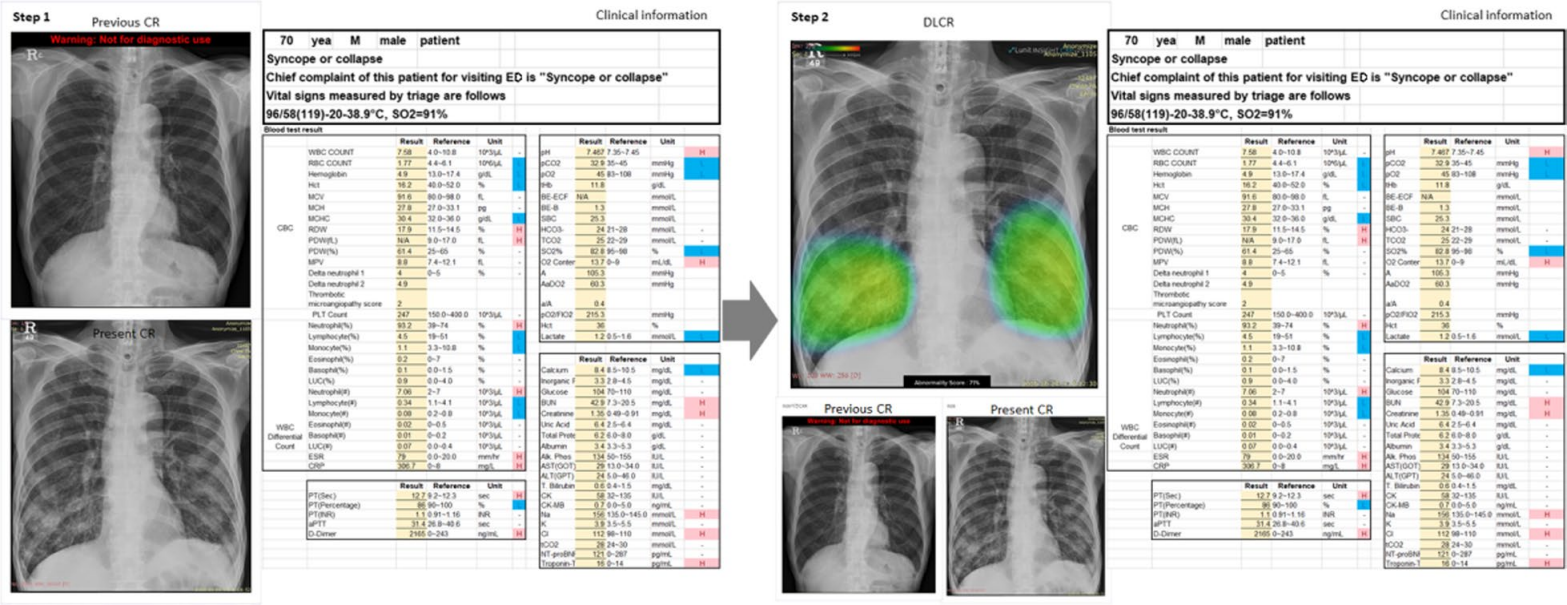
Representative case for performance assessment. (Left) CRs and the patients’ clinical and demographic characteristics were presented to the participating ED physicians in the first step. (Right) In the second step, the same information was presented, although the assessment was made using DLCR. CR, chest radiograph; ED, emergency department; DLCR, deep learning-based assistive technology on CR interpretation

Simulation sessions in this study were designed based on the study site’s process for patient management in the ED. Performance assessment was conducted with each participant in a separate room and under a researcher’s supervision. Participants were asked to interpret CR findings presented alongside the patients’ demographic and clinical characteristics (age, sex, chief complaint, vital signs, and laboratory test results at admission). CRs in the anteroposterior and posteroanterior views were provided; in cases where previous CRs were available, they were provided at the same time. Clinical information of the patient and performed CRs were provided to the participants through a monitor screen. The simulation session comprised two steps, each of which was recorded through a web-based form (Google forms; Google, Mountain View, CA). In the first step, participants were asked to examine the given CR for abnormalities and to make a clinical decision regarding patient disposition based on the provided clinical information without DLCR. In the second step, participants performed simulation on the same case after being added the DLCR from the first step. At this stage, participants were not allowed to modify their responses provided in the first step; all responses were recorded in real-time. There was no time limit for the participant to complete the simulation (Fig. 1).

### Deep learning algorithm

All CR data used in the present study were analysed using commercially available DLCR (Lunit INSIGHT for Chest Radiography, version 4.7.2; Lunit; available at http://insight.lunit.io), approved by the Korea Food and Drug Administration for clinical use. This algorithm was developed to detect three major abnormalities (lung nodule, consolidation, and pneumothorax), with suspicious lesions within target regions displayed as a heat map with the corresponding abnormality scores provided as percentage values at the bottom of the image. The abnormality score represents the maximum probability value estimated for the finding of a lung nodule, consolidation, or pneumothorax, as determined by the programme itself. A single performance test of the algorithm using CR data from 244 patients was previously conducted by the Korea Food and Drug Administration, with a reported AUROC of 0.994 (95% CI 0.987–1.000), sensitivity of 97.26%, and specificity of 92.86%. The probability score for the high-sensitivity cut-off used in this test was 0.15. The deep learning algorithm receives a CR as input and then generates a heat map. This algorithm is composed of 120 convolutional layers with four dense blocks [21] and three parallel classifiers for each abnormality. In the training stage, the algorithm was trained to classify abnormal CRs and localize the region of the abnormalities. Two types of loss functions were used to train the algorithm: classification loss and localization loss. Given an input CR with annotations for the lesion location, the loss function for each abnormality was defined as the sum of the classification loss and localization loss. The classification loss was defined as the binary cross-entropy between the label of CR and the max-pooling of the corresponding probability map. The localization loss was defined as the average pixel-wise binary cross-entropy between the annotation on CR and the corresponding probability map. The losses of the three abnormalities were then finally summed to form the final loss function. In the case of CR inputs without annotation, only the classification loss was utilized. To predict lesion location, even without location information, a weakly-supervised localization scheme was used to train the classification loss. All of the hyperparameters were initialized randomly via Gaussian distribution and optimized using the stochastic gradient descent (SGD) with a mini-batch size of 64. A learning rate of 0.01 and a momentum term of 0.9 was used to stabilize the training, and the learning rate decreased from 0.01 to 0.001 after 30 epochs. The models were trained up to 40 epochs.

### Definition of the reference standard

The reference standard for the presence of a thoracic abnormality was defined retrospectively. One board-certified emergency radiologist with 12 years of experience independently reviewed the CRs and same-day chest CT scans along with any available previous imaging findings and other clinical information, such as that provided in medical records (including laboratory findings). Subsequently, a reviewer determined whether a given CR presented radiological evidence of an abnormality in any of the algorithm’s target lesions. In the absence of an interval change between consecutive CRs, the findings were considered ‘normal’.

### Performance assessment in participants

Participant performance was assessed based on the following tasks. The first task involved detecting abnormalities on CRs. If none of the target abnormalities were detected by the participants, then their response to the task was marked as normal. Subsequently, the participants were asked to provide a clinical decision for patient disposition, based on their CR findings and other relevant information. Participants were asked to select their recommendations from the following: (1) clear impression for ED disposition; (2) impression not clear but additional tests not required, patient eligible for discharge; (3) impression not clear and additional evaluation required at an outpatient clinic; and (4) impression not clear and additional evaluation required, patient recommended for ED admission.

### Outcome measures

The primary outcome was the change in the CR interpretation performance by the same ED physician. The secondary outcome was the consistency of clinical decision-making by the same ED physician.

### Statistical analyses

Categorical variables are presented as counts and percentages; between-group differences were examined with the chi-square test. For continuous variables, the normality assumption was verified using the Shapiro–Wilk test; the variables that satisfied this assumption are reported as means (standard deviations), and the corresponding between-group differences were tested with Student’s t-test. The remaining continuous variables are presented as medians (Q1, Q3), and the corresponding between-group differences were tested with the Mann–Whitney U test. CR interpretation performance was assessed in terms of sensitivity, specificity, accuracy, and AUROC. These indexes were calculated separately for each participant and combined for all participants. The consistency in clinical decision-making was assessed with the kappa statistic; A k value of less than 0.20 was defined as minor agreement; a k value of 0.21–0.40 was defined as fair agreement; a k value of 0.41–0.60 was defined as moderate agreement; a k value of 0.61–0.80 was defined as high agreement; and a k value greater than 0.80 was defined as excellent agreement [22]. Within-participant comparison of AUROC estimates was performed with the DeLong test; between-participant comparison of AUROC estimates was performed using the multi-reader multi-case (MRMC) ROC method. Comparisons of sensitivity, specificity, and accuracy parameters were performed with the generalised estimating equation method. The kappa statistics were compared using the bootstrap method. Findings were considered statistically significant at *P* values of < 0.05. A P value < 0.05 was considered to indicate a statistically significant difference between two groups in all analyses. All analyses were conducted using SAS, version 9.4 (SAS Institute), and R, version 3.6.3 (The R Foundation for Statistical Computing).

### Patient and public involvement

Patients or the public were not involved in the design, or conduct, or reporting, or dissemination plans of our research.

## Results

Demographic and clinical characteristics of the included patients are summarised in Table 1. A defined CR abnormality was noted in a total of 259 patients (66.8%). In addition, in 274 patients (70.6%), a previous CR was available; a CR in the anteroposterior view was available in 189 patients (48.7%).

**Table 1:** Demographic and clinical characteristics of patients included as simulation cases

Variable	Total (n = 388)	Normal (n = 129)	Abnormal (n = 259)
Age, years	68 (58, 77)	62 (44, 72)	70 (62, 78)
Male	222 (57.22%)	65 (50.39%)	157 (60.62%)
Previous CR available	274 (70.62%)	84 (65.12%)	190 (73.36%)
View of CR			
Anteroposterior	189 (48.71%)	46 (35.66%)	143 (55.21%)
Posteroanterior	199 (51.29%)	83 (64.34%)	116 (44.79%)
Abnormality score	33.80 (5.74, 68.75)	3.35 (1.57, 8.89)	59.3 (31.77, 76.25)

Continuous variables are expressed as medians (Q1, Q3). Categorical variables are expressed as counts (%)

CR, chest radiograph

Changes in the CR interpretation performance of ED physicians, stratified by DLCR use, are presented in Table 2. There was a significant difference in the overall AUROC for CR interpretation among ED physicians working with DLCR when compared to those working without DLCR (*P* < 0.001). Other performance indices, including sensitivity, specificity, and accuracy of CR interpretation, in overall ED physicians were also significantly different, depending on DLCR use (*P* < 0.001, 0.015, < 0.001 respectively). After using DLCR, the sensitivity and accuracy of detecting abnormalities on CRs increased significantly in all ED physicians, while the AUROC values increased significantly except for those of one board-certified ED physician.

**Table 2:** Changes in the CR interpretation performance by DLCR use

	Without DLCR	With DLCR	*P* value	Without DLCR	With DLCR	*P* value
	Sensitivity (95% CI)			Specificity (95% CI)		
Physician 1	61.00 (55.06, 66.94)	66.80 (61.06, 72.53)	0.001	96.90 (93.91, 99.89)	94.57 (90.66, 98.48)	0.177
Physician 2	65.64 (59.85, 71.42)	72.97 (67.56, 78.38)	< 0.001	87.60 (81.91,93.29)	87.60 (81.91, 93.29)	> 0.999
Physician 3	56.76 (50.72, 62.79)	64.86 (59.05, 70.68)	< 0.001	91.47 (86.65, 96.29)	96.12 (92.79, 99.46)	0.012
Physician 4	67.18 (61.46, 72.90)	76.06 (70.87, 81.26)	< 0.001	82.95 (76.46, 89.44)	86.05 (80.07, 92.03)	0.344
Physician 5	71.43 (65.93, 76.93)	75.68 (70.45, 80.90)	0.004	81.40 (74.68, 88.11)	86.05 (80.07, 92.03)	0.054
Physician 6	62.93 (57.05, 68.82)	69.88 (64.30, 75.47)	0.005	86.05 (80.07, 92.03)	93.02 (88.63, 97.42)	0.026
Physician 7	49.03 (42.95, 55.12)	55.98 (49.94, 62.03)	0.002	91.47 (86.65, 96.29)	96.12 (92.79, 99.46)	0.031
Overall physicians	62.00 (55.64, 68.35)	68.89 (64.68, 73.10)	< 0.001	88.26 (82.90, 93.62)	91.36 (88.49, 94.23)	0.015
	Accuracy (95% CI)			AUROC (95% CI)		
Physician 1	72.94 (68.52, 77.36)	76.03 (71.68, 80.28)	0.022	0.790 (0.756, 0.823)	0.807 (0.772, 0.842)	0.167
Physician 2	72.94 (68.52, 77.36)	77.84 (73.70, 81.97)	0.003	0.766 (0.726, 0.807)	0.803 (0.764, 0.842)	0.032
Physician 3	68.30 (63.67, 72.93)	75.26 (70.96, 79.55)	< 0.001	0.741 (0.702, 0.780)	0.805 (0.771, 0.839)	< 0.001
Physician 4	72.42 (67.98, 76.87)	79.38 (75.36, 83.41)	< 0.001	0.751 (0.707, 0.794)	0.811 (0.771, 0.850)	0.004
Physician 5	74.74 (70.42, 79.07)	79.12 (75.08, 83.17)	< 0.001	0.764 (0.721, 0.808)	0.809 (0.769, 0.848)	0.002
Physician 6	70.62 (66.09, 75.15)	77.58 (73.43, 81.73)	< 0.001	0.745 (0.703, 0.787)	0.815 (0.779, 0.850)	< 0.001
Physician 7	63.14 (58.34, 67.94)	69.33 (64.74, 73.92)	< 0.001	0.703 (0.664, 0.742)	0.761 (0.726, 0.795)	< 0.001
Overall physicians	70.73 (65.91, 75.54)	76.36 (73.21, 79.51)	< 0.001	0.751 (0.719, 0.783)	0.801 (0.774, 0.828)	< 0.001

CR, chest radiograph; DLCR, deep learning-based assistive technology for chest radiograph; CI, confidence interval; AUROC, area under the receiver operating characteristic curve

Sensitivity and accuracy estimates were significantly different between physicians who did and did not use DLCR, regardless of their level of experience; meanwhile, the AUROC and specificity values showed significant differences only in the inexperienced ED physician group (Table 3).

**Table 3:** Changes in CR interpretation performance stratified by emergency physicians’ work experience

Variable		Inexperienced physician	Experienced physician
Sensitivity (95% CI)	Without DLCR	62.65 (55.88, 69.41)	61.13 (53.65, 68.62)
	With DLCR	69.40 (65.14, 73.66)	68.21 (63.46, 72.97)
	*P* value	< 0.001	< 0.001
Specificity (95% CI)	Without DLCR	85.47 (79.09, 91.84)	91.98 (86.06, 94.01)
	With DLCR	90.31 (87.21, 93.41)	92.76 (89.56, 95.96)
	*P* value	0.004	0.577
Accuracy (95% CI)	Without DLCR	70.23 (65.05, 75.40)	71.39 (65.79, 76.98)
	With DLCR	76.35 (73.17, 79.53)	76.37 (72.83, 79.91)
	*P* value	< 0.001	< 0.001
AUROC (95% CI)	Without DLCR	0.741 (0.701, 0.780)	0.766 (0.721, 0.811)
	With DLCR	0.799 (0.761, 0.837)	0.805 (0.780, 0.829)
	*P* value	< 0.001	0.079

CR, chest radiograph; DLCR, deep learning-based assistive technology for chest radiograph; CI, confidence interval; AUROC, area under the receiver operating characteristic curve

**Fig. 2:**
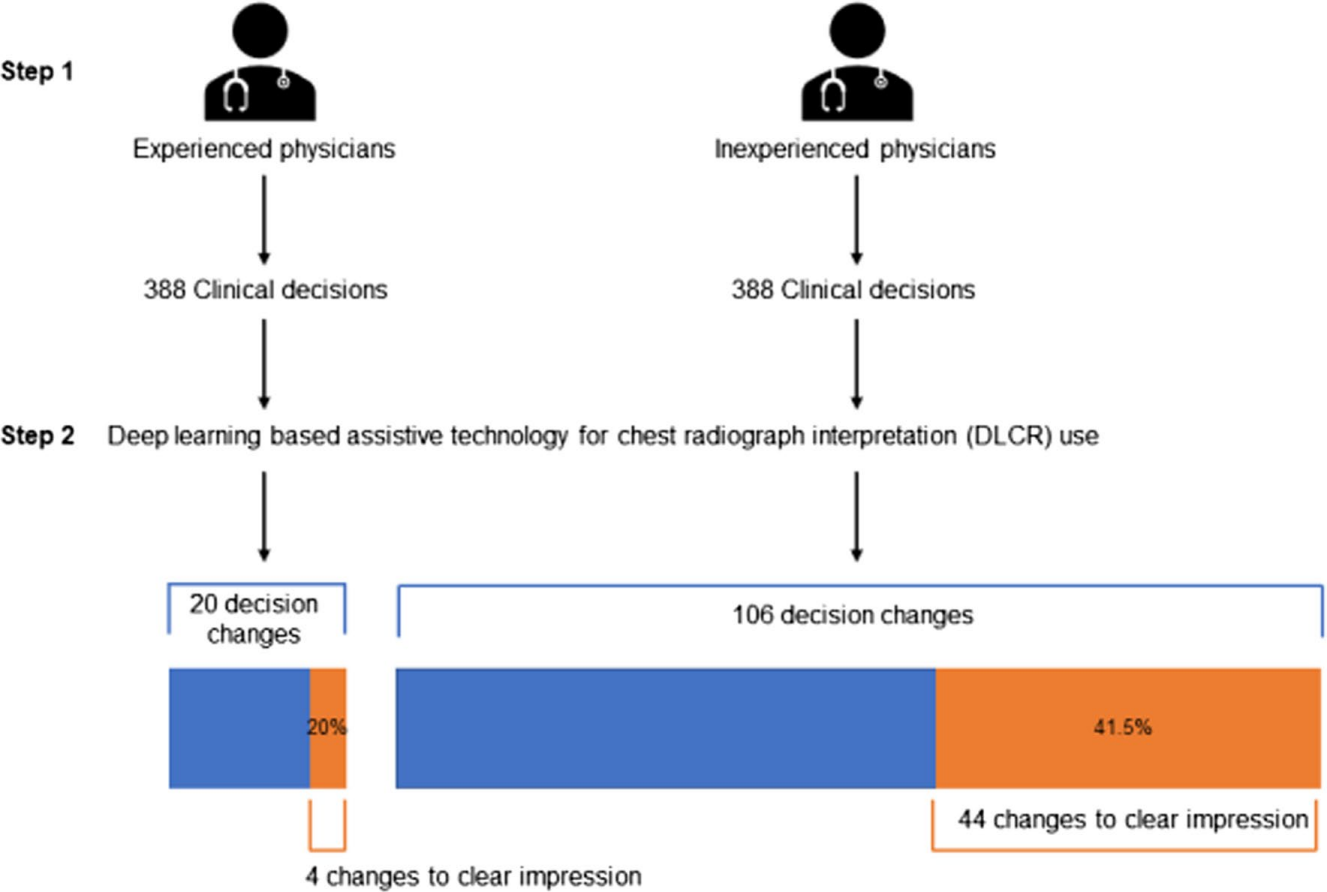
Flowchart of changes in clinical decisions after assessing CRs with deep learning-based assistive technology. CR, chest radiograph

Table 4 summarises the findings on clinical decision-making consistency, according to DLCR usage. The overall kappa value was 0.902 (95% CI 0.884–0.920); the corresponding values for the experienced and inexperienced groups were 0.956 (95% CI 0.934–0.979) and 0.862 (95% CI 0.835–0.889), respectively; these estimates were significantly different (*P* < 0.001). Overall, a total of 126 clinical decisions changed after using DLCR. Of these, 48 decisions were changed from ‘unclear’ to ‘clear’ impression for ED disposition. These kinds of changes in clinical decisions were significantly more frequent among inexperienced physicians than among experienced physicians (Fig. 2) (*P* = 0.026).

**Table 4:** Consistency in clinical decision-making by ED physicians according to DLCR use

Physician		Kappa value		*P* value
Experienced physicians	Physician 1	0.954 (0.920, 0.988)	0.956 (0.934, 0.979)	< 0.001
	Physician 2	0.964 (0.930, 0.999)		
	Physician 3	0.957 (0.929, 0.985)		
Inexperienced physicians	Physician 4	0.794 (0.736, 0.852)	0.862 (0.835, 0.889)	
	Physician 5	0.970 (0.941, 0.999)		
	Physician 6	0.862 (0.792, 0.933)		
	Physician 7	0.807 (0.754, 0.860)		
Overall physicians		0.902 (0.884, 0.920)		

ED, emergency department; DLCR, deep learning-based assistive technology for chest radiograph

## Discussion

In the present study, the use of DLCR improved ED physicians’ interpretations of CRs in a clinical setting. Moreover, our findings indicate that the use of DLCR significantly improved the sensitivity to CR abnormalities regardless of the interpreting physician’s experience. This finding corroborates those from previous studies on DLCR use [16, 17, 19]. In addition, although the use of DLCR did not significantly affect clinical decisions made by experienced physicians at the ED, it did affect those made by inexperienced physicians; specifically, the use of DLCR improved inexperienced physicians’ confidence in their clinical judgement.

ED physicians make decisions based on multiple variables along with CR findings [23]; they tend to place more weight on the overall clinical assessment than on CR findings alone. Nevertheless, the results of the present study suggest that compared with experienced physicians, inexperienced physicians might rely more on DLCR, as it might increase their confidence in their own clinical judgement. Because interpreting CRs or integrating the clinical information associated with CRs is relatively difficult for inexperienced physicians, it is likely that the influence of DLCR use on clinical decision-making is more frequently observed among inexperienced physicians than among their experienced counterparts. Moreover, chest radiography tends to be performed to assess whether further imaging studies are required to confirm the diagnosis in the ED [17]; as such, using DLCR, which helps screen for abnormalities rather than establish a diagnosis, is practical. This study showed that DLCR use can improve the sensitivity of CR abnormality detection by physicians.

Globally, emergency care resources are limited, particularly in rural areas [24], where EDs often lack imaging equipment such as CT or magnetic resonance imaging scanners [25]. In this context, the ability to accurately interpret X-ray findings, when available, is paramount to effective patient care [26]. Moreover, hospitals in under-resourced areas also have restricted staff, whereby a single physician is responsible for the entire department instead of several physicians being on duty simultaneously [23]. Our study findings suggest that the use of DLCR can support CR interpretation performed by ED physicians, particularly those who are less experienced or under time and resource constraints; this technology might be used effectively in low-resource regions. Previous studies on automatic detection algorithms mainly examined their diagnostic performance [19, 20, 27]. In particular, after COVID-19 era, these algorithms are expected to play a useful role in decision-making in clinical practice [28, 29]. However, for this technology to be used in clinical practice, it must demonstrate technical superiority in addition to usefulness to the end user, for example, a physician [30]. To the best of our knowledge, this is the first study to examine the influence of DLCR use on changes in clinical decisions made by ED physicians. To reflect real-life practice, this study provided participants with information on the patients’ previous CRs, chief complaint, vital signs, and laboratory test results at ED presentation, all of which are considered in clinical practice alongside CR findings.

This study has some limitations that should be considered when interpreting its findings. First, because this study was a simulation-based trial, it did not accurately represent real-world practice. In this simulation, findings from a physical examination and ultrasound and those from intensive history taking could not be included; thus, they were not considered in the decision-making. Second, because of the limitation of the target range of DLCR used in this study, other abnormalities identified on CRs could not be verified; further research with an algorithm that involves a broader target range is required. Third, the changes in clinical decisions reported in the present study were not equivalent to improved clinical outcomes considering the cost–benefit of DLCR. Future studies should examine the effectiveness of DLCR considering cost–benefit on patient outcomes in the real-world setting. Lastly, the possibility of selection bias exists because participants were recruited only from physicians working in the same ED. Especially, recommendations for clinical decision presented as options in the simulation cannot be generalized for all ED physicians.

## Conclusions

In conclusion, the present study demonstrated that use of DLCR would improve the CR interpretation performance of ED physicians; in addition, the use of DLCR affects clinical decisions made by inexperienced physicians. Further studies are required to validate DLCR use in a real-world setting before this technology is included in routine clinical practice.

## Data Availability

The datasets generated during the current study are available from the corresponding author on reasonable request.

## Abbreviations

AUROC: Area under the receiver operating characteristic curve
CI: Confidence interval
CR: Chest radiograph
CT: Computed tomography
DLCR: Deep learning-based assistive technology
ED: Emergency department
